# Exploring the evolution of facial feminization and masculinization surgery: a bibliometric analysis and visualization study

**DOI:** 10.1186/s40902-024-00424-x

**Published:** 2024-06-05

**Authors:** Omer Uranbey, Omer Faruk Kaygisiz, Ferhat Ayrancı, Saim Yanik

**Affiliations:** 1 Department of Oral and Maxillofacial Surgery, Faculty of Dentistry, Ordu University, Ordu, Turkey; 2 Department of Oral and Maxillofacial Surgery, Faculty of Dentistry, Gaziantep University, Gaziantep, Turkey

**Keywords:** Facial feminization surgery, Facial masculinization surgery, Mapping analysis, Bibliometric analysis

## Abstract

**Background:**

This study aims to conduct a bibliometric analysis of the current literature related to facial feminization surgery (FFS) and facial masculinization surgery (FMS) to understand the patterns, trends, and evolution of research topics. In addition, it aims to objectively identify the important articles that constitute the primary backbone of the FFS/FMS literature and provide a resource for education and new studies in this emerging field.

**Results:**

Using the principles of the Leiden Manifesto, 384 publications from the Web of Science from 1987 to 2023 were analyzed. The analysis included cross-country collaboration, keyword trends, affiliations, co-citation networks, and clustering**.** The results showed an increasing trend in FFS/FMS publications, with the USA leading in both publications (*n* = 238) and citations (*n* = 2420). The most cited journal was the Journal of Plastic and Reconstructive Surgery. The results indicate a high growth rate, with an H-index of 34 and an average citation of 11.41 per article. Co-occurrence analysis revealed evolving keywords such as “forehead” (*n* = 52) and “quality of life” (*n* = 44). The timeline view illustrated the terms reflecting current areas of interest such as #patient satisfaction and #gender-affirming care.

**Conclusion:**

The study reveals the influence of countries, institutions, authors, and emerging trends, supporting the anticipation that FFS/FMS will be a critical field of study in the future. The findings contribute to understanding the global landscape of FFS/FMS research, facilitating informed decision-making for researchers, and clinicians in the field of maxillofacial surgery.

## Background

In a world where the boundaries of self-expression are constantly expanding, the field of maxillofacial surgery is also evolving to meet different demands. Cosmetic surgery in this field has become a powerful tool to harmonize the physical appearance with the mental structure of the individual [1]. The face plays an important role in gender recognition by oneself and others, and its features are associated with perceived gender [2]. The term “facial feminization surgery” (FFS) includes a combination of hard and soft tissue surgeries designed to transform a masculine face into a feminine face, while the term “facial masculinization surgery” (FMS) is used as a term that includes a combination of surgeries designed to transform a feminine face into a masculine face [3].

Surgical interventions for gender dysphoria were traditionally based on genital contouring until the mid-1980s when Dr. Douglas Ousterhout pioneered FFS, a groundbreaking approach to addressing the facial differences between the male and female sexes [4]. In this context, craniomaxillofacial surgeons have sought to identify clinical differences between male and female facial skeletons through objective anthropological measurements [5]. As a result of the investigations, it was determined that the soft and hard tissues including the glabellar, orbital, nasal, mental, and cervical regions contain secondary sex characteristics and constitute significant differences between male and female individuals [5]. The male facial structure tends to be generally angular and sharply contoured, while females have oval and delicate facial structures with broader angular transitions [6]. Although soft tissue features and fat distribution play an important role in the facial appearance of both sexes, the major differences are primarily a result of the bone structures underneath [7].

In order to achieve characteristic gender appearances, surgical operations such as frontal sinus reduction cranioplasty, supraorbital shaping, hairline positioning on the scalp, orbital repositioning for intercanthal distance, eyebrow positioning are possible in the upper third of the face, and rhinoplasty, mandibular gonial angle changes, genioplasty, malar adipose tissue operations, cheek implants, lip shaping and tracheal shaping are possible in the lower and mid-face third [8, 9].

These surgeries, which are also performed cosmetically on individuals regardless of gender reassignment, provide a great field of study for examining the transformative effects on individuals in search of authenticity and self-affirmation [1].

While interest in surgical procedures such as FFS and FMS, which can play an important role in alleviating gender dysphoria, has increased over time, there has been no objective evaluation of the literature surrounding these major and complex surgeries [10].

Unlike a systematic literature review, bibliometric analysis is an analytical method used to obtain formal and quantitative data on the current state of a field, and it facilitates the monitoring of academic trends through visualization software [11, 12]. It can be used as a preparatory stage for a systematic literature review. The primary goal of the bibliometric approach is to obtain quantitative data and numerical measurement indicators about research performance. The interpretation of these metrics provides quantitative insights into the productivity of countries, authors, universities and journals, weak and strong research areas, literature gaps, collaborative networks, and the widespread impact of the results produced in the field [13].

In this study, a bibliometric and scientific mapping analysis of the disorganized literature in the field of FFS and FMS aimed to provide researchers and clinicians with valuable information about the patterns, trends, and evolution of research topics and to identify current views and possible future research focuses. In addition, by analyzing citations in research sources, it is planned to make comparisons in terms of author contributions and global collaboration capabilities.

## Methods

### Study design

This study is in accordance with the principles of the Leiden Manifesto, is exempt from institutional review board approval as a bibliometric analysis, uses publicly available sources, does not generate new data, and does not have access to private patient information [14]. Articles were retrieved from the Web of Science Core Collection (WoSCC) database on the same day (December 28, 2023) to avoid bias due to daily database updates. The literature was filtered for the time span between 1987 and 2023.

### Data collection

Medical Subject Headings (MeSH) were used to select the following search terms: “ALL=(facial feminization) OR ALL=(facial masculinization) OR ALL=(facial feminization surgery) OR ALL=(forehead contouring) OR ALL=(facial gender affirmation surgery) OR ALL=(facial gender affirmation surgery)”. No language restrictions were applied. Document types other than original articles and reviews were excluded from the analysis. The filtering was done manually for the 723 documents found as a result of the search. In the filtering process; studies that included surgeries performed in the facial region for feminization and masculinization, described the operation techniques/processes/complications for these surgeries, and presented facial secondary sex characteristics were included. Studies outside the oral and maxillofacial surgery discipline, describing dermatologic and injection-based treatments, and describing surgeries performed for deformities were excluded. A list of articles was created on a Microsoft Excel document (Microsoft Corporation, Redmond, USA) and the following information was recorded: journal name, ranking by number of citations, citation density (number of citations per year), first author’s name, year of publication, first author’s institution and country of origin, study type, study design, research areas, keywords, author keywords, index.

### Data analyses

The finalized data were imported into the R Studio (version 4.3.2, J.J. Allaire, MA, USA), Citespace (version 6.2.R6, Drexel University, USA), and VOS-viewer (Leiden University, The Netherlands) for relevant statistical computing and generation of maps and graphics. CiteSpace and VOS-viewer were used to examine the relationships between keywords, authors, institutions, countries, and collaborating teams across regions. The top 10 keywords and the top 10 cited authors with the most significant citation bursts were identified by Citespace. A manual comparison was performed between the number of citations on Web of Science and Google Scholar. This comparison was performed on the same day to avoid bias. Co-citation analysis was used to identify common focus and hot research topics.

## Results

### Overall results

**Fig. 1:**
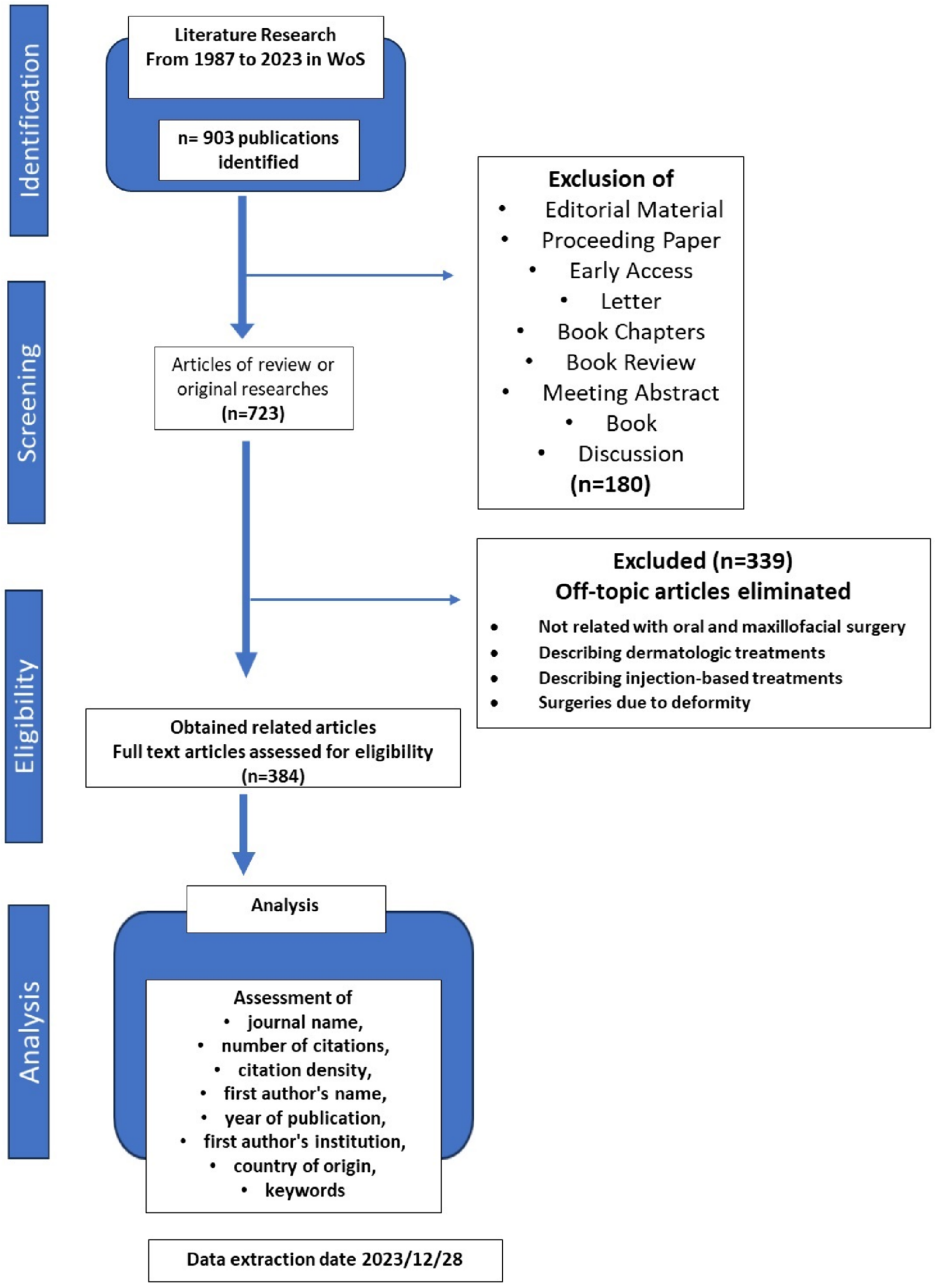
Flowchart of the articles analyzed in the study

**Fig. 2:**
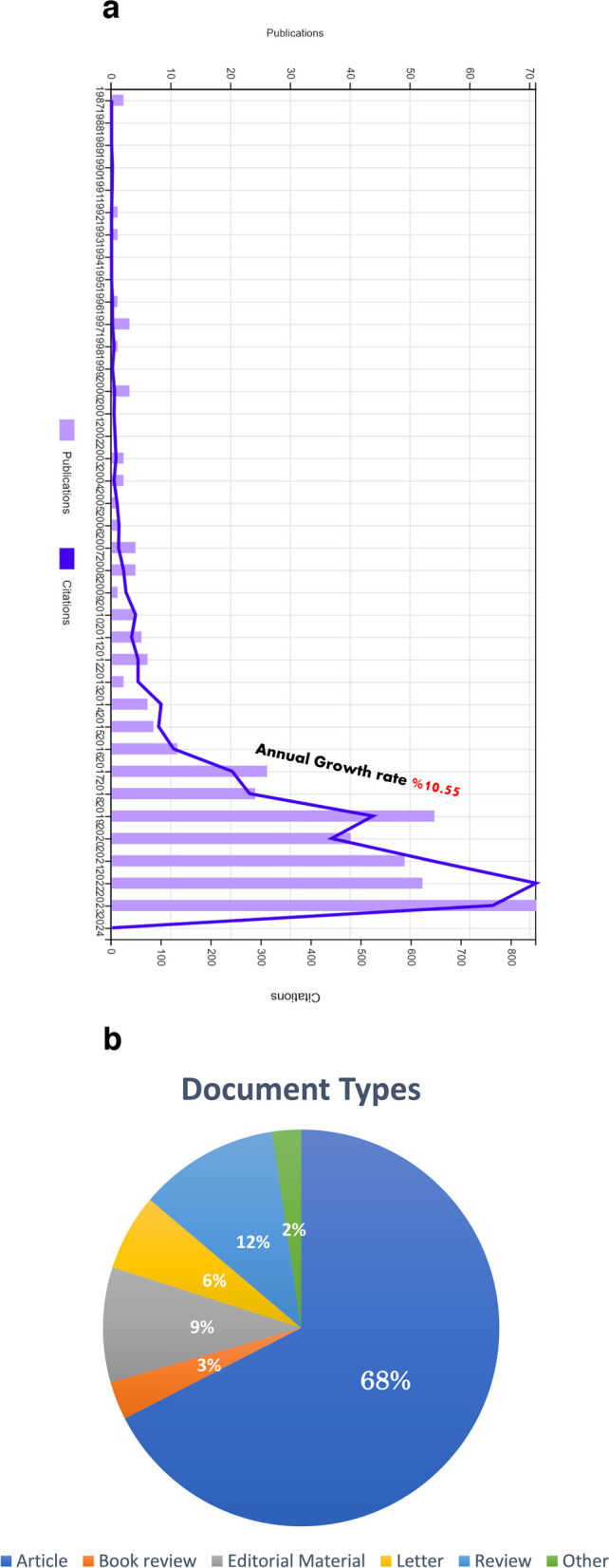
**a** FFS/FMS publications (light purple) and its relationship with the number of citations (blue) by year. Taken from Web of Science Journal Citation Reports (JCR). **b** Pie chart of document types related to FFS/FMS literature from 1987 to 2023

In the first survey, 903 articles were obtained. After manual content filtering, the number was reduced to 384 (Fig. 1). The included articles received citations from a total of 2292 other studies, involving 2003 unique authors and 289 self-citations. In total, there were 4382 citations, with 2458 coming from authors other than the included articles. The average number of citations of the included articles is 11.41 and the H-index is 34. With an annual growth rate of 10.55%, the number of global publications is increasing every year (Fig. 2a). The majority of the publications were original articles (68%), followed by reviews (12%) and editorial material (9%) (Fig. 2b). The Journal of Plastic and Reconstructive Surgery published the most articles on this topic (*n* = 52, highest H-index = 18).

### Cross-country collaboration

**Fig. 3:**
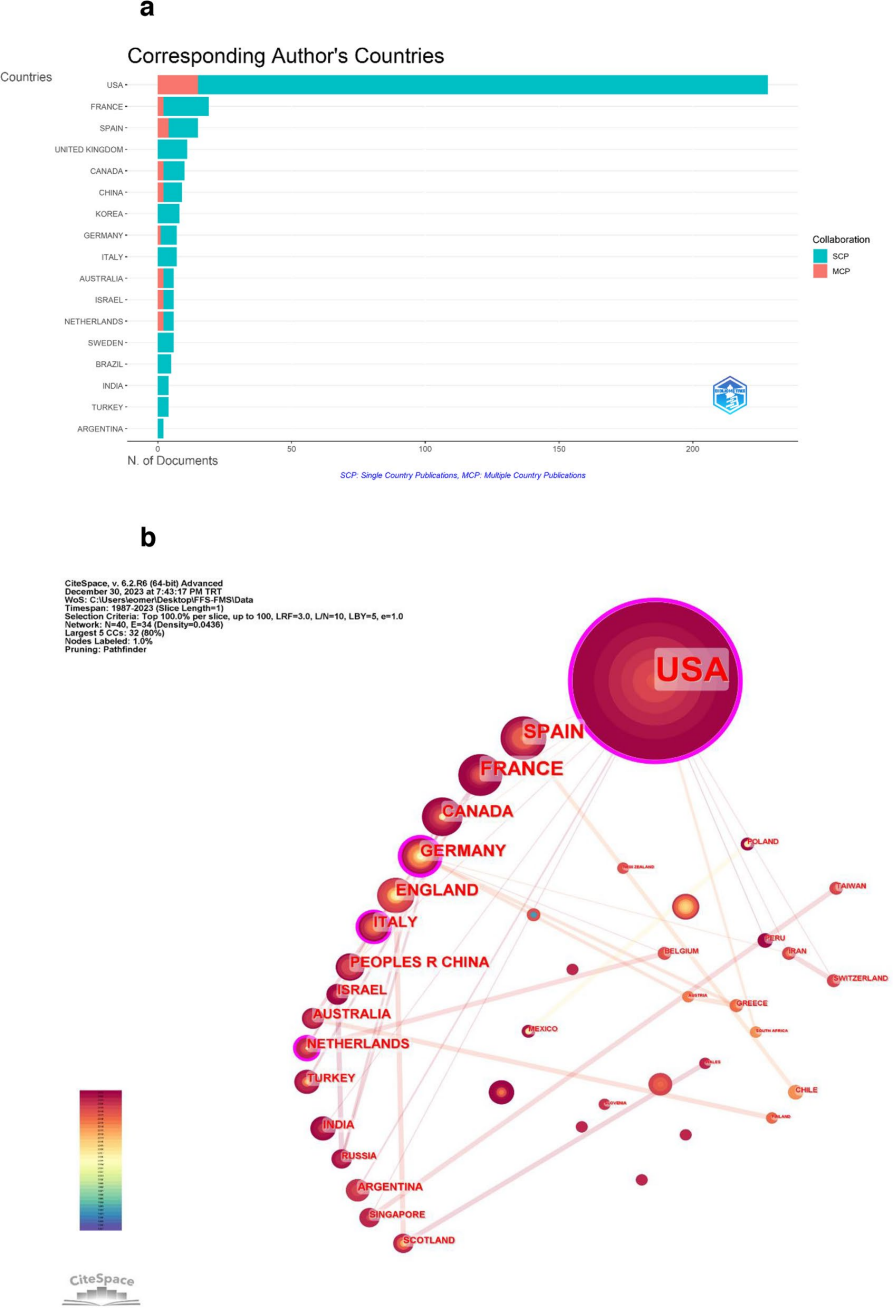
**a** Corresponding author’s countries**. b** Visualization of cross-country collaboration

Citespace and R Studio were used together to evaluate the collaboration between countries. The United States contributes the most to global research (*n* = 238) with the most citations (*n* = 2420). The ranking of countries by the number of documents published was as follows: the USA (*n* = 228), France (*n* = 19), Spain (*n* = 15), UK (*n* = 11), with the highest international collaboration observed in the USA (Multiple Country Publications “MCP” = 15) (Fig. 3a).

A total of 40 nodes and 34 links were identified through Citespace. The size of the nodes indicates the frequency of co-citation, and the links between nodes indicate the connections between co-citations. Circle colors represent the time of co-citation to these countries (Fig. 3b). The top five countries in terms of centrality (purple circle) are the USA (0.43), Germany (0.36), the Netherlands (0.15), Italy (0.11), and Spain (0.06).

### Keyword and co-occurrence analysis

**Fig. 4:**
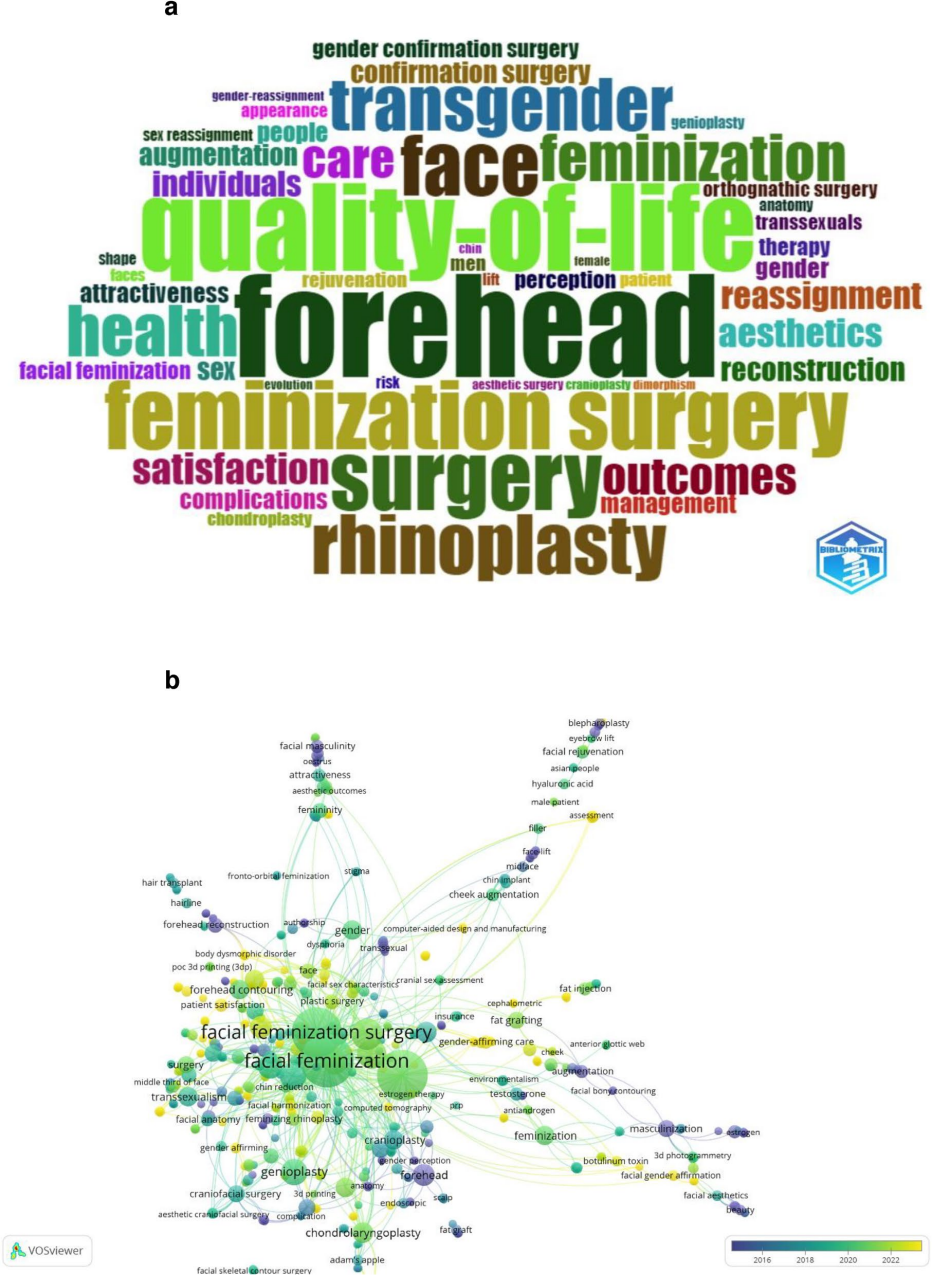
**a** WordCloud illustration based on KeyWords Plus. **b** Visualization of co-occurrence analysis

The WordCloud illustrates a network of keywords (Fig. 4a). Among the keywords extracted from the titles of the cited articles (KeyWord Plus), the distribution around the most important keyword “forehead” (*n* = 52) can be observed. Other important KeyWord Plus terms are “quality-of-life, feminization surgery, rhinoplasty”, each with a frequency of “30” or more. The size of the words in the font underscores how often the keyword is mentioned, while their central placement highlights their significance in the broader context.

Co-occurrence analysis is used to examine the relationship between keywords proposed by authors, regarding the frequency and repetition of their occurrences. Constructing a co-occurrence network of keywords, as shown in Fig. 4b, allows us to explore the conceptual structure of the domain under study. The minimum number of occurrences of a keyword chosen as “1” resulted in 597 keywords, 42 clusters, 2122 links, and 2404 total link strength was found. On the timeline, purple keywords appeared earlier than green and yellow keywords appeared later.

### Co-citation analysis

**Fig. 5:**
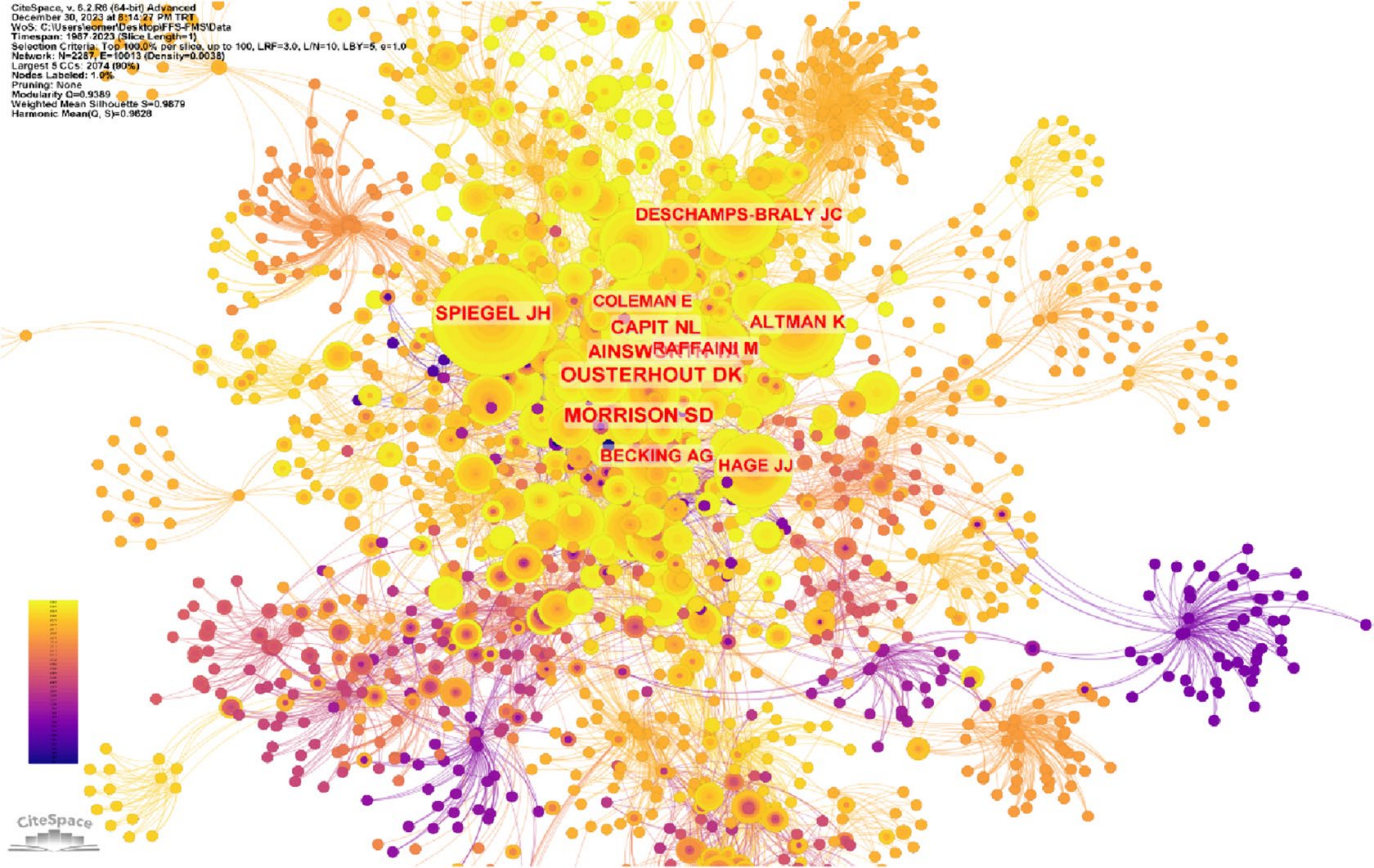
This figure illustrates the co-citation network pertaining to FFS/FMS publications

**Fig. 6:**
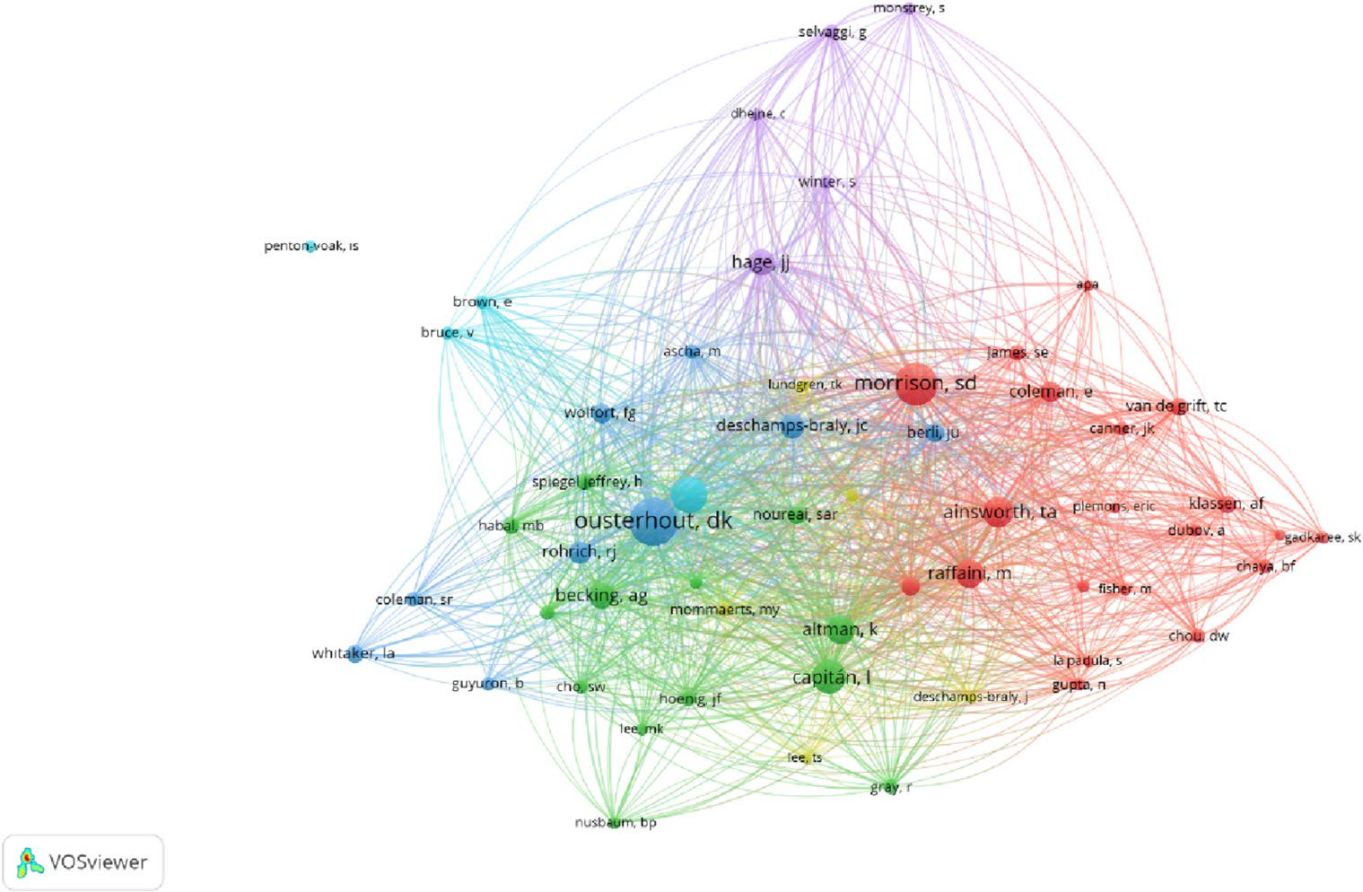
Mapping of the co-cited authors on FFS/FMS

In this visual representation, the nodes correspond to the cited references, while the links between the nodes indicate shared citation relationships (Fig. 5). Following the completion of co-citation analysis, a total of 2287 nodes and 10,013 connections were identified. The most cited publication was Ainsworth and Spiegel, with 224 citations [15] (Table 1). The largest radius reference (most co-cited publication) in the network was that of Ousterhout DK, with 148 co-citations [5]. A total of 57 authors with a minimum of 15 citations were analyzed using VOS viewer. According to the linkage between authors and co-citations, 6 clusters, 1317 links, and 14,213 total link strength are formed (Fig. 6). It can be seen that the strongest links are grouped around 4 authors; Ousterhout, DK (total link strength = 2556 times), Morrison, SD (total link strength = 2614 times), Becking, AG (total link strength = 1039 times) and Hage, JJ (total link strength = 1231 times). The top 10 articles with the most citations in different databases (Web of Science and Google Scholar) are summarized in Table 1.

**Table 1:** Top 10 most cited articles by order of WS citations

Ranking	Reference	Year	Journal	Country	WS	CD	GS
1	Ainsworth et al. [15]	2010	Qualıty of Lıfe Research	USA	224	16	338
2	Ousterhout, DK [5]	1987	Plastıc and Reconstructıve Surgery	USA	143	3864	221
3	Morrison et al. [24]	2016	Plastıc and Reconstructıve Surgery	USA	127	15,875	174
4	Gangestad et al. [32]	2003	Evolutıon and Human Behavıor	USA	121	5761	259
5	Altman K [8].	2012	Internatıonal Journal of Oral and Maxıllofacıal Surgery	UK	111	925	181
6	Spiegel JH [33]	2011	Laryngoscope	USA	110	8461	154
7	Grift et al. [34]	2018	Journal of Sex & Marıtal Therapy	The Netherlands	108	18	184
8	Cieri, et al. [35]	2014	Current Anthropology	USA	101	10.1	270
9	Raffaini et al. [36]	2016	Plastıc and Reconstructıve Surgery	Italy	86	14,333	117
10	Capitan et al. [37]	2014	Plastıc and Reconstructıve Surgery	Spain	84	8.4	118

*WS* Web of Science database number of citations, *CD* citation density (number of citations per year), *GS* Google Scholar number of citations

### Institutional collaboration analysis

**Fig. 7:**
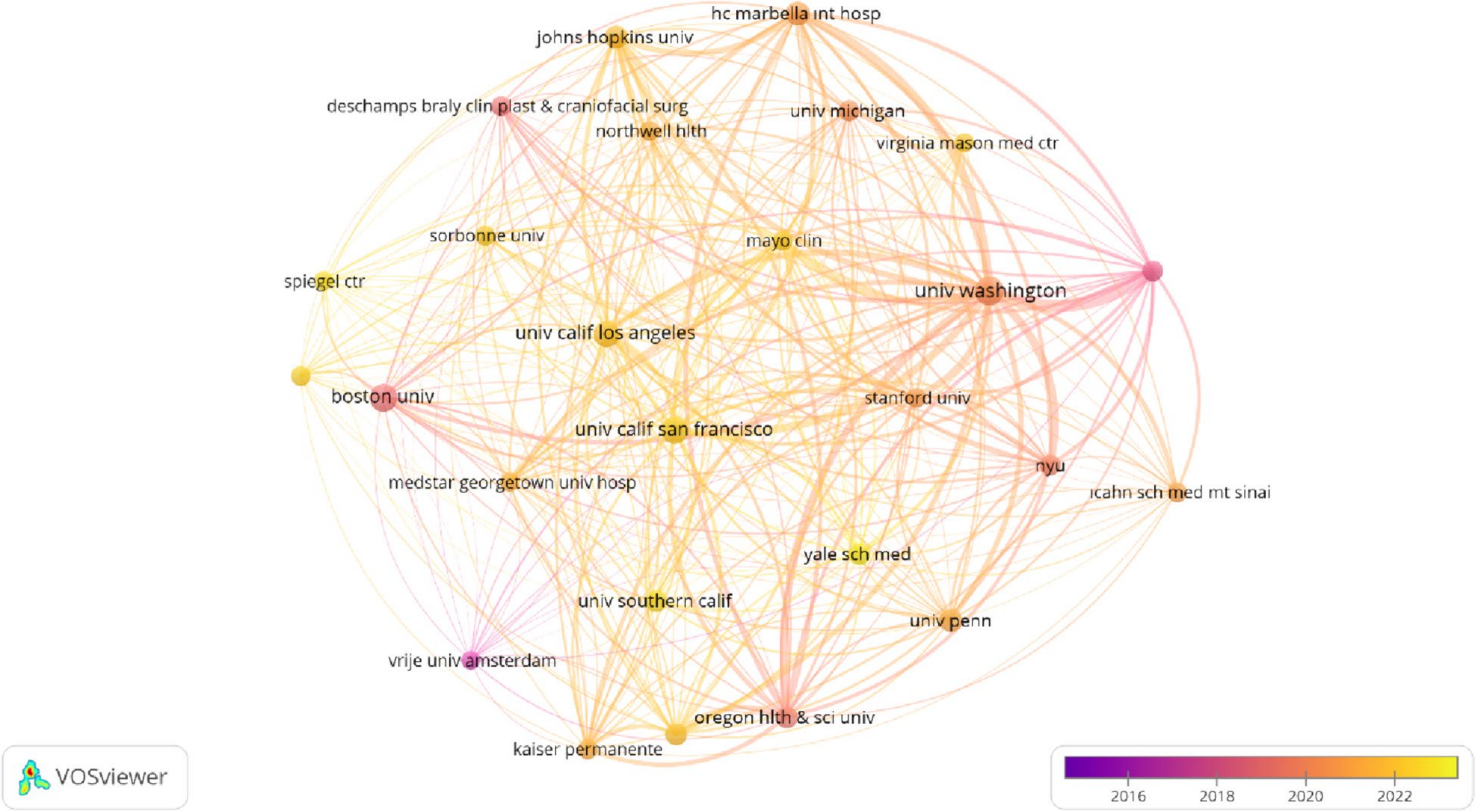
Visualization by bibliographic coupling of the institutions

Institutions with at least 5 documents and at least 5 citations were selected as search criteria. The annual view of 26 institutions was visualized, forming a total of 4 clusters, 325 links, link strength of 35,793 (Fig. 7). The top 5 institutions with the highest total link strength were shown as follows: University of Washington (total link strength = 9465 times), University of California, San Francisco (total link strength = 4692 times), HC Marbella International Hospital (total link strength = 4539 times), Brownstein and Crane Surgical Services (total link strength = 4314 times), and Oregon Health and Science University (total link strength = 4206 times).

### Cited author clustering analysis

**Fig. 8:**
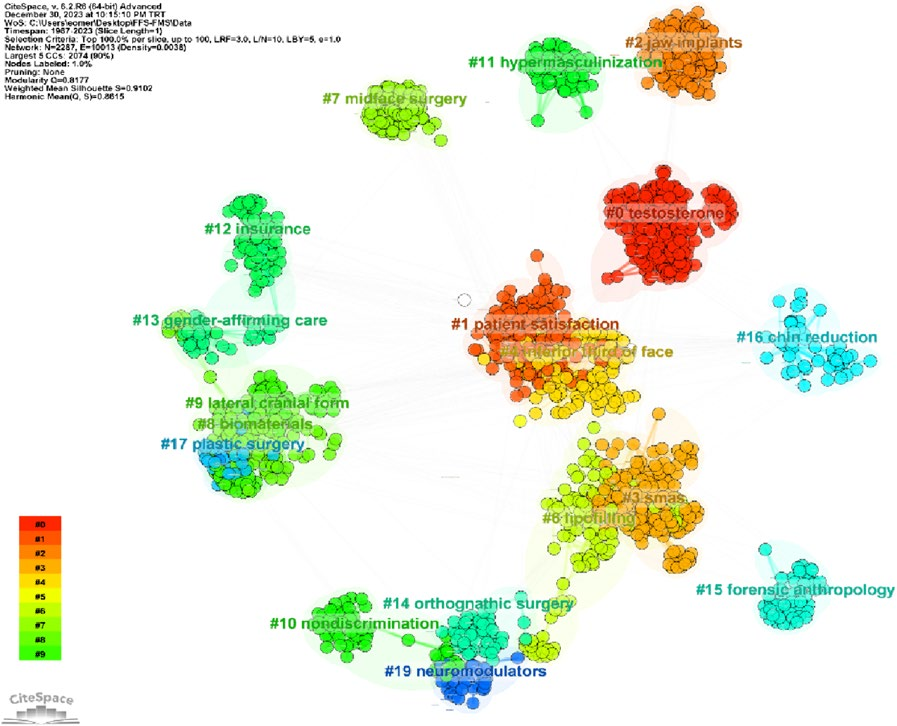
A mapping of the cluster analysis of cited authors is shown. As a result, the analysis shows that the FFS/FMS literature is organized into 19 clusters. The clusters are labeled from 0 to 19, from largest to smallest

**Fig. 9:**
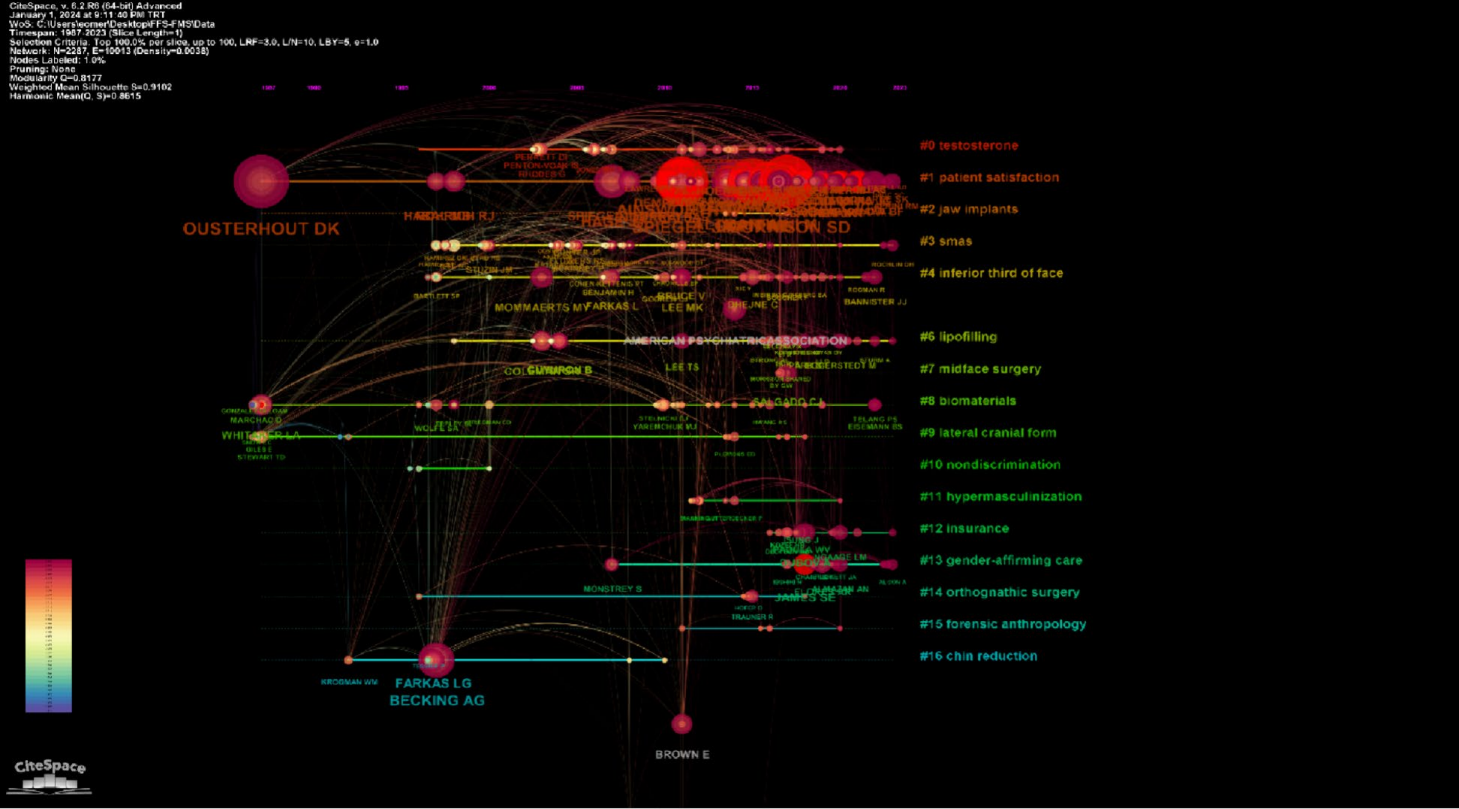
The timeline format of cluster analysis

The cluster analysis is shown in Fig. 8. The timeline format of the cluster analysis is shown in Fig. 9. As a result, the analysis shows that the FFS/FMS literature is divided into 19 clusters. The clusters are labeled from 0 to 19, from largest to smallest. The cluster labels were determined according to log-likelihood ratio (LLR), Latent Semantic Indexing (LSI), and mutual information (MI). The largest cluster was related: “testosterone (cluster #0, size = 222, silhouette = 0.938), patient satisfaction (cluster #1, size = 180, silhouette = 0.618) and jaw implants (cluster #2, size = 174, silhouette = 1).” When the time period view of the clusters is analyzed, the publications with a citation explosion are marked as bright red circles.

### Emerging topics from burst analysis

**Fig. 10:**
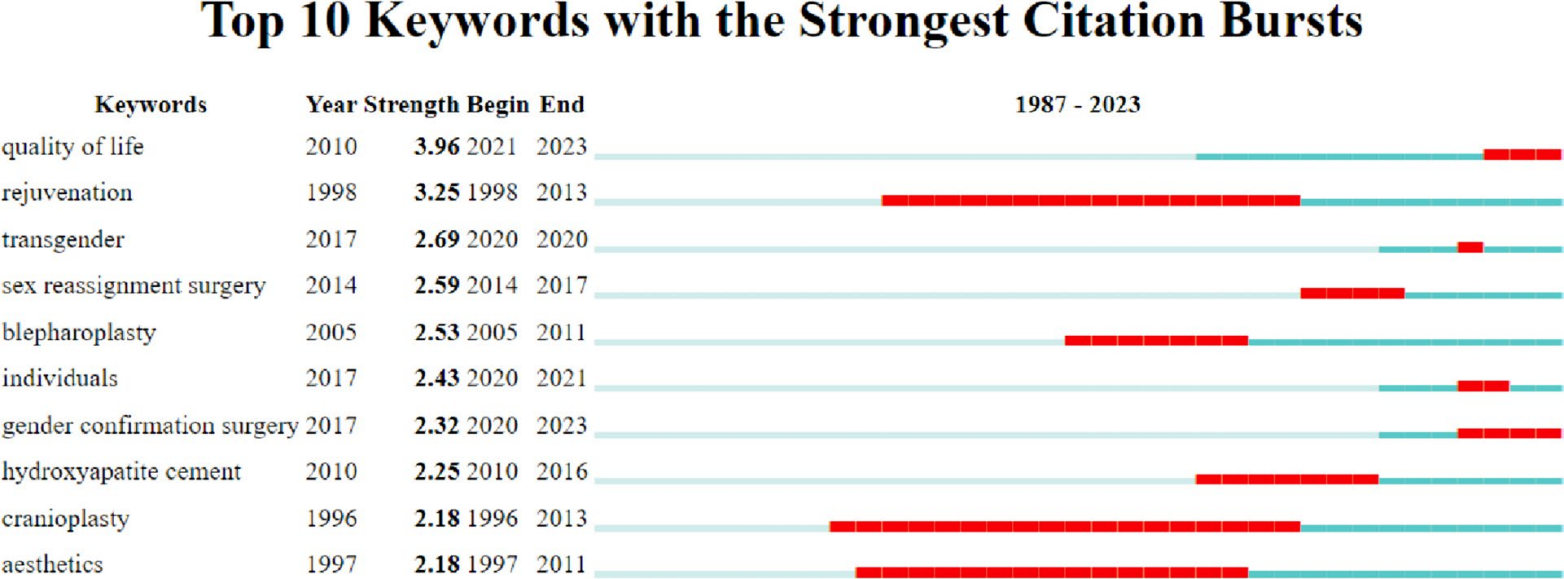
Top 10 keywords with bursts of citations in articles on FFS/FMS publications from 1996 to 2021 are highlighted. The time period is represented by a blue line, while the beginning and end of the time interval of each burst keyword are represented by a red line

The top 10 keywords with the strongest citation bursts in the scientific literature were analyzed and visualized in the bursts map (Fig. 10). The lines in red stood for the burst detection years. Keywords with red lines extending to the latest year can indicate the research hotspots in a short period of time in the future. The most significant latest burst keywords include “quality of life” (2021–2023), “gender confirmation surgery” (2020–2023) and “individuals” (2020–2021). The highest citation burst was “quality of life” (strength = 3.96) and had a duration of 2 years. Thus, studies on these aspects could indicate potential trends and possible frontiers of the FFS/FMS field.

## Dıscussıon

Scientific articles are in the past tense from the time of their publication, but citations to these articles serve as the source for subsequent publications. By performing a detailed analysis of the scientific literature in a given field, it is possible to follow the progress of the subject over time and predict the future path of the literature [13]. A bibliometric analysis was conducted to determine the evolution and trends in the FFS/FMS-related literature. Mapping methods were used to make this theoretical analysis comprehensible. This study represents the most comprehensive bibliographic analysis of the FFS/FMS literature to date and can provide readers with evidence-based information on the evolution, major research areas, and directions.

The number of FFS-FMS publications in international journals has increased since 2016. In particular, there is a two-fold increase in 2017 compared to 2016 and in 2019 compared to 2018. The launch of “Transgender Health” in 2016, the first peer-reviewed journal addressing the health needs of transgender individuals, may be effective in this increase. After a pause in the rate of increase between 2020 and 2022, the number of publications of FFS/FMS literature peaked in 2023. It is believed that this short-term pause is a reflection of the COVID pandemic, as in all other selective surgical branches, and that the authors have turned their focus to studies aimed at this global pandemic [16]. When analyzing the general data, a very high annual growth rate of 10.55% was found in the literature. There are studies that show gender confirmation surgeries have tripled in the last 10 years in the USA alone [17]. The general data of the study support our thesis that FFS/FMS will be an important topic of discussion in the future.

The USA has contributed most to this prominence in the literature, both in terms of publications (*n* = 238) and citations (*n* = 2420). It is also the country with the most interaction in terms of international cooperation. It is followed by countries such as France (*n* = 19), Spain (*n* = 15), UK (*n* = 11). All of these countries are in the high-income category according to World Bank 2023 data [18]. There is a dramatic difference in the quantity and quality of contributions to the literature between the USA and other countries in the same development category. This situation shows that interest and awareness of surgeries are influenced by local social perceptions, such as local interactions and country-based trends. On the other hand, the USA has witnessed a rise in annual cases in recent years due to changes in federal and state laws mandating insurance coverage for gender-affirming surgeries [17]. General data suggest that gender dysphoria increases with economic development.

The keywords revealed by the WordCloud are used to guide future research and identify milestones. In such a visualization, the font size, color code, and distance of words from other keywords provide clues about the frequency and importance of that keyword and other topics with which it may co-occur. The most prominent keyword is “forehead” (*n* = 52), which is considered one of the most critical regions in FFS-FMS operations. Many commonly held opinions suggest that the morphometry of the upper third of the face plays a crucial role in determining facial beauty [19]. The preoperative analysis and planning of the upper third of the face, which exhibits distinctive features between the male and female sexes, is also of great importance [19]. While forehead contouring surgery is not a novel subject, discussions about the severity of complications arising from procedures in this area and strategies to mitigate these morbidities are still among the popular topics [20]. Other prominent keywords are “quality of life” (*n* = 44), “outcomes” (*n* = 19), “satisfaction” (*n* = 16), “complications'” (*n* = 10). In the practice of FFS/FMS, it is possible to see the implications of these words that stand out in the WordCloud. There is a lack of long-term follow-up data on FFS/FMS procedures, which often involve multiple levels of surgery and long operating times [21]. The overall recovery time, which is quite long in this transformative process for many individuals, may increase further depending on complications [22]. While FFS/FMS aims to align an individual's physical appearance with their gender identity, the psychological adjustment to the new facial features can be challenging. Unrealistic expectations or dissatisfaction with the results may lead to emotional distress or regret. Recently, however, to reduce all these morbidities, attempts have been made to improve surgical outcomes with techniques called “all-in-one F-FFS”, in which many procedures are performed simultaneously [23]. Moreover, multidisciplinary teams, in which a more comprehensive level of care can be provided to these individuals and the treatments can be performed in a single center, provide a higher level of satisfaction and are effective in reducing overall costs [22].

Ousterhout, the author who stands out in the co-citation analysis, is a pioneer in this field with the first and most important study of facial feminization in the international FMS-FFS literature [5]. This study, which established the basics of facial feminization, explained the importance of the upper third of the face in the feminine appearance and became a guiding source for subsequent studies. Despite Ousterhout’s publication in 1987, the fact that the most cited publication was by Ainsworth et al. 23 years later shows that this was a groundbreaking publication in the FFS-FMS literature. This comprehensive study examined how the quality of life was affected in 247 transgender individuals who underwent facial feminization and sex reassignment surgery [15]. As a result, they found that transgender women had lower mental health-related quality of life compared to the general female population. Surgical interventions, such as FFS, were identified as factors contributing to the improvement of mental health-related quality of life. Ousterhout [5], Morrison [24], and Becking [25] are among the most cited authors and are at the forefront of publications on FFS-FMS operation. Becking et al. studied psychosocial functioning in facial surgery to facilitate surgical treatment planning [25]. This highly cited study is one of the key works of literature that shows that the subjective satisfaction of patients who undergo an FFS procedure alone is not an indicator of the ultimate success of the procedure. This study, which advocates the standardization of facial differences using objective methods, will continue to shape the literature.

The four institutes with the highest institutional affiliation strength (University of Washington, University of California, Brownstein and Crane Surgical Services, Oregon Health and Science University) are located in the USA, indicating that the USA is the central country in the FFS-FMS literature. One of the facilities (HC Marbella International Hospital) is owned by Spain. Convincingly, Spain is one of the countries that has contributed to the change in social tolerance in this way [26]. From a social and constitutional point of view, the anti-discrimination Zerolo Law approved in 2022 and the fact that it is the country that is the most supportive of transgender rights in a study carried out in 2016, puts Spain in a different position in this regard compared to other European countries [27]. The authors of this article believe that Spain's potential in the FFS/FMS literature can be further developed. The Vrije Universiteit Amsterdam is among the institutions where important work has been done, but the most active research on the topic is in the USA.

The silhouette metric is used to assess the uncertainty related to the determination of the type of cluster [28]. This value ranges from “− 1” to “1”, indicating uncertainty in interpreting cluster type, with 1 denoting excellent isolation [29]. In this study, the overall silhouette value was “0.9106”, indicating excellent separation within the FFS/FMS literature. Among these clusters, Cluster #2 (LLR: behavioral modernity LSI: social tolerance MI: jaw implants) exhibited the highest silhouette value (size = 174, silhouette = 1). Treatment of gender dysphoria is not limited to surgery, and there are indications where hormonal regulation of testosterone and estradiol is sufficient [30]. Especially in individuals who do not consider surgical treatment or who are not suitable for surgical treatment, hormone therapies are currently applied [31]. The #1 patient satisfaction cluster, one of the clusters with the highest citation burst, emphasizes the importance of a patient-based perspective in success. Success is an indicator of the patient’s satisfaction with the results as well as the perfection of medical and surgical interventions. Therefore, in the future, the appropriate patient selection and individualized treatment protocols will focus on the patient's expectations as much as on the success of the surgery.

To our knowledge, this is the first study that attempts to analyze outcomes related to FFS/FMS procedures using a science mapping approach.

Limitations of our study include the fact that data were only available from the Web of Science database. The choice of a database to provide data for mapping software may lead to the omission of publications in other databases (e.g., Scopus). Another limitation is that the current bibliometric analysis includes a limited number of articles and may lead to sharp divergences. However, by analyzing such a rapidly growing area of surgery, our study will provide a resource for future research.

## Conclusıons

Indicators reflected in the field such as the changing prejudices of societies, the development of global health systems, the spread of multidisciplinary centers, and the increase in the coverage of health insurance are increasing in line with the general outputs of our bibliometric literature study; it is seen that it will support the desire of individuals to undergo surgery for feminization and masculinization and we think that the FFS/FMS literature will be a very important field of study in the future.

## Data Availability

Datasets used and/or analyzed in this study are available from the corresponding author.

## Abbreviations

FFS: Facial feminization surgery
FMS: Facial masculinization surgery
WosCC: Web of Science Core Collection
USA: United States of America
UK: United Kingdom
LLR: Log-likelihood ratio
LSI: Latent Semantic Indexing
MI: Mutual ınformation
